# Indoxyl sulfate induces apoptosis in mononuclear blood cells via mitochondrial pathway

**DOI:** 10.1038/s41598-023-40824-z

**Published:** 2023-08-28

**Authors:** Anna Pieniazek, Joanna Bernasinska-Slomczewska, Pawel Hikisz

**Affiliations:** Department of Oncobiology and Epigenetics, Faculty of Biology and Environmental Protection, University of Lodz, Ul. Pomorska 141/143, 90-236 Lodz, Poland

**Keywords:** Cell biology, Diseases

## Abstract

The consequence of chronic kidney disease is the accumulation of metabolic products called uremic toxins in the body. Indoxyl sulfate (IS) is a toxin with a high affinity for proteins. This study focuses on the deleterious effect of IS, especially apoptosis induction, in mononuclear blood cells (MNCs). Thus, in MNCs treated with IS at three different concentrations for 24 h, the survival, mitochondrial potential, caspases activity and expression, Bcl-2 and Bax protein expression, DNA damage, and PARP degradation were estimated. The study showed a decrease in survival and mitochondrial potential of MNCs treated with IS compared to the control. IS increased the activity of caspase 2-, 3-, 9-, and the expression of caspase 3-, and 9- in MNCs but does not affect the activity of caspase 6- and 8. The treatment of MNCs with IS also increased DNA damage and degradation of PARP. Indoxyl sulfate significantly influences the expression of Bcl-2 and Bax proteins. Indoxyl sulfate induces the programmed death of MNCs through the intrinsic mitochondrial apoptotic pathway. The observed cellular changes are mostly dose-dependent.

## Introduction

Chronic kidney disease (CKD) is associated with impaired renal excretory function and, consequently, the accumulation of uremic toxins in the body. Uremic toxins that originate from cellular metabolism are classified as water-soluble and low molecular weight compounds with high protein-bound affinity^1^. Several protein-binding uremic toxins, including indoxyl sulfate, p-cresol sulfate, and hippuric acid, are produced by gut bacteria and are hard to remove by current conventional dialysis^2^.

Indoxyl sulfate (IS) is a toxin with a strong affinity for proteins. About 97% of the pool of this compound is bound to proteins in the body^2^. Such modification of proteins may impair their function and, consequently lead to cell metabolic disorders. Accumulation of this toxin in patients with chronic kidney disease (CKD) can reach 80 times the concentrations observed in healthy individuals (30–53 mg/L in CKD and 0.5–0.6 mg/L in healthy people)^3,4^. Regarding chemical structure, IS belongs to indoles with an aromatic heterocyclic structure. In the human body, this compound may be formed as a result of tryptophan metabolism. The indolic pathway of tryptophan metabolism leads to the formation of IS and indole-3-acetic acid (IAA)^5^. Both of these metabolic products and intermediates (kynurenine, quinolinic acid, and kynurenic acid) have been classified as protein-binding uremic toxins^3^.

It was shown that high levels of IS have pro-inflammatory and pro-oxidative properties, contributing to the development and progression of comorbidities in patients with CKD^6^. It has been described that inflammation in CKD is the critical factor contributing to the development of cardiovascular disease, atherosclerosis, anemia, and erythropoietin resistance^7,8^. Many factors, such as current oxidative stress and high concentrations of uremic toxins or other disorders, can trigger inflammation in patients with CKD. Moreover, inflammation, protein and DNA damage, and other factors play an essential role in inducing apoptosis in cells.

Additionally, it is assumed that indoxyl sulfate is involved in the induction of oxidative stress. Formation of reactive oxygen species (ROS) in human renal tubular epithelial cells (HK-2)^9^, erythrocytes^10^, lymphocytes^11^, human umbilical vein endothelial cells (HUVEC)^2^, and intestinal epithelial cells (IEC-6)^12^ has been observed under the influence of IS. The imbalance between the amount of reactive oxygen species and the antioxidant system results in strong oxidative stress that damages cellular components. The consequence of these damages may be the induction of programmed cell death. Some studies show the activation of pro-apoptotic factors in cells exposed to IS^13–15^.

In our work, we hypothesized that IS might induce apoptosis in mononuclear blood cells. We investigated whether indoxyl sulfate affects the processes of apoptosis induction and DNA damage in mononuclear blood cells. Due to this, we evaluated caspase expression and activity, mitochondrial potential, and DNA fragmentation in the cells treated with IS at three concentrations chosen on the basis of available scientific reports.

## Results

**Figure 1:**
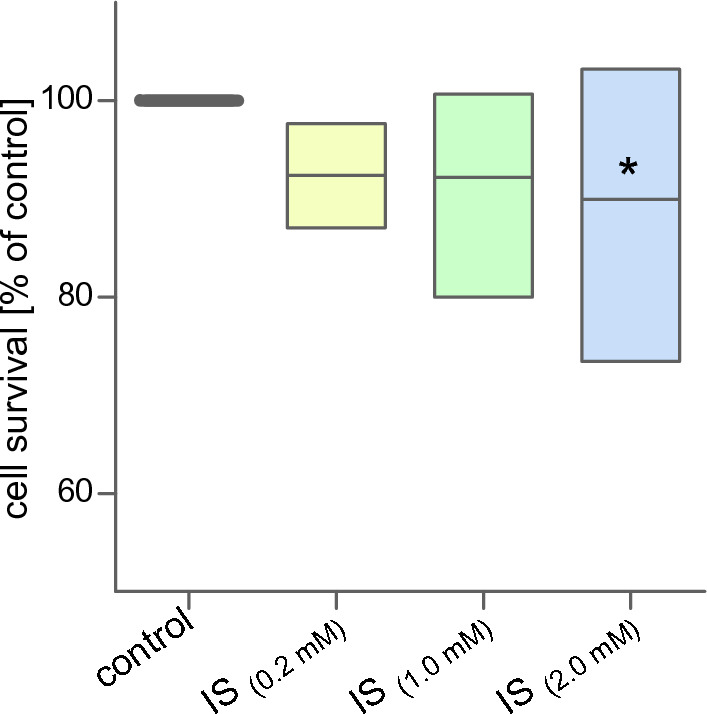
The survival of human mononuclear blood cells after 24 h of treatment with indoxyl sulfate. Each column represents cells from 8 different individuals (n = 8). Data were presented as mean with a box of minimum and maximum values, and the survival of control cells were arbitrarily taken as 100%, *p < 0.05—IS_(2.0 mM)_ versus control.

Isolated mononuclear blood cells were exposed to three concentrations of indoxyl sulfate (0.2, 1.0, and 2.0 mM) for 24 h. The cytotoxicity of indoxyl sulfate on MNCs was compared to untreated (control) cells, in which survival was arbitrarily taken as 100% (Fig. 1). Neutral red, a dye staining lysosomes in living cells, was used to assess cytotoxicity. The results indicated that indoxyl sulfate did not cause significant changes in MNCs survival at concentrations of 0.2 and 1.0 mM. A statistically significant decrease in cell survival was observed after 24 h treatment with 2.0 mM of indoxyl sulfate. The decrease in survival of cells treated with IS at a concentration of 2.0 mM was about 10% compared to control cells.

**Figure 2:**
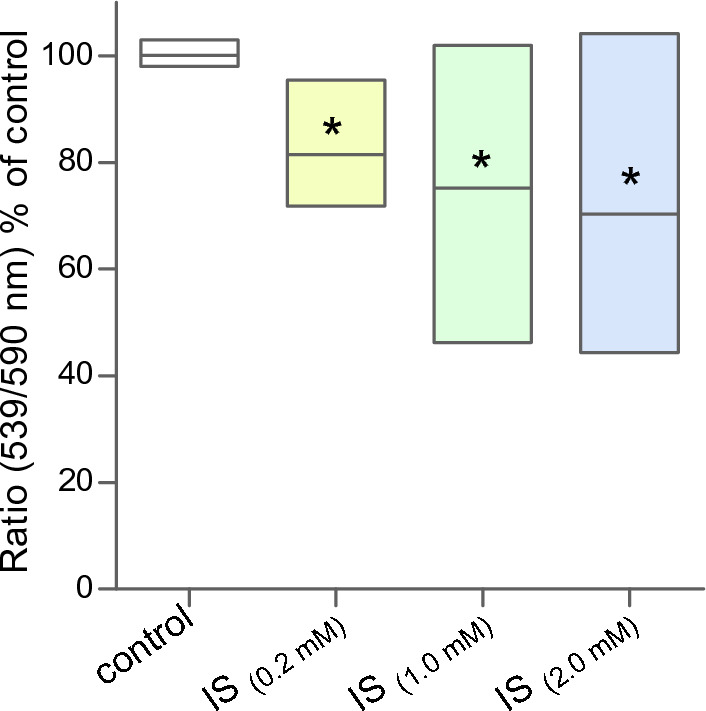
Changes in mitochondrial potential in MNCs treated with indoxyl sulfate were measured immediately after treatment. Each column represents cells from 10 different individuals (n = 10). Data were presented as mean with box of minimum and maximum values, a mean ratio (539/590 nm) in control cells was arbitrarily taken as 100%, *p < 0.05—IS_(0.2 mM)_, IS_(1.0 mM)_, and IS_(2.0 mM)_, versus control.

The biochemical activity of cells is mainly dependent on properly functioning mitochondria. Usually, it is the ability of the mitochondria to make ATP appropriately in response to energy demands. Therefore, the mitochondrial membrane potential was another parameter estimated in MNCs treated with indoxyl sulfate. The results showed that indoxyl sulfate significantly reduces the mitochondrial potential in MNCs (Fig. 2). The data were expressed as a percentage of the change in the ratio (530 nm/590 nm). A significant decrease in the mitochondrial potential, by about 19% compared to the control, was observed after incubation of cells with indoxyl sulfate at a concentration of 0.2 mM. The increase in the concentration of indoxyl sulfate to which the cells were exposed resulted in a further decrease in the mitochondrial potential. Thus, after treating the cells with IS at a concentration of 2.0 mM, this parameter was reduced to 70% compared to the value of control cells (100%).

**Figure 3:**
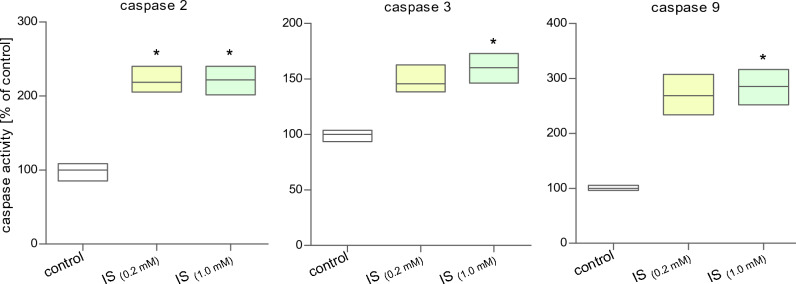
Detection of caspases 2, 3, and 9 activity in MNCs incubated with indoxyl sulfate. Each column represents cells from 5 different individuals (n = 5). Data were presented as a median with a box of minimum and maximum value, * *p* < 0.05—IS_(0.2 mM)_, IS_(1.0 mM)_, and IS_(2.0 mM)_, versus control.

In the next step of our work, we evaluated the caspases activity in cells incubated with indoxyl sulfate. The activity of caspase 2, 3, 6, 8, and 9 was estimated. The results showed a significant increase in caspase 2 activity in MNCs incubated with IS at a concentration of 0.2 mM and 1.0 mM (Fig. 3). In turn, caspase 3 and 9 significantly increased in cells treated with IS at a concentration of 1.0 mM. In the case of cells incubated with a lower concentration of IS (0.2 mM), the increase in the activity of these caspases was statistically insignificant. Caspase 6 and 8 activity in MNCs exposed to IS were also assessed; however, no statistically significant changes in activity were observed for these caspases (Table 1).

**Table 1:** Caspase 6 and 8 activity in lysates of MNCs treated with indoxyl sulfate.

	Caspase activity (% of control)			Significant differences
	Control	IS (0.2 mM)	IS (1.0 mM)	
Caspase 6	98.4 (92.9; 114.8)	101.1 (95.6; 106.6)	109.3 (95.6; 117.5)	Not significant
Caspase 8	96.2 (89.9; 115.1)	96.2 (89.9; 115.1	115.1 (93.1; 124.6)	Not significant

The data were presented as median with minimum and maximum values. The median represents cells from 5 different individuals (n = 5).

**Figure 4:**
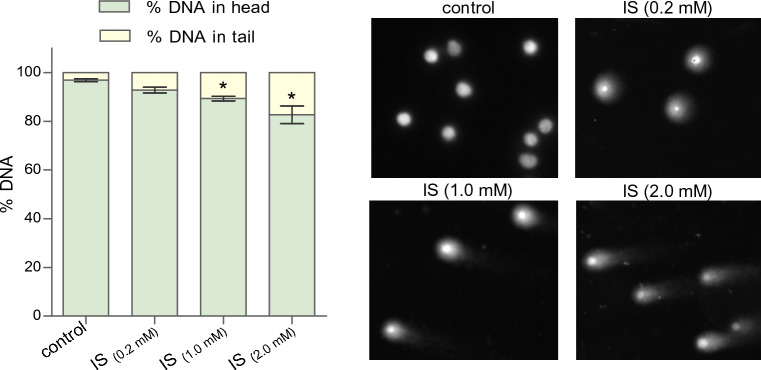
The percent of DNA in comet head and tail of mononuclear blood cells after treatment with indoxyl sulfate. Each column represents cells from 6 different individuals (n = 6). Data were presented as mean ± SD, * *p* < 0.05—IS_(1.0 mM)_, and IS_(2.0 mM)_, versus control.

The degree of DNA damage in cells exposed to indoxyl sulfate was also assessed. The comet assay is a good indicator for the evaluation of genotoxicity. A significant increase of single-stranded and double-stranded DNA breaks was found in cells treated with IS at a concentration of 1.0 mM and 2 mM (Fig. 4). In MNCs treated with IS (0.2 mM), the increase of DNA damage was about 4% in comparison to control value. For cells treated with 1.0 mM and 2.0 mM of IS, the increase in single-stranded and double-stranded DNA breaks averaged 7.5% and 14.2%, respectively, compared to controls.

**Figure 5:**
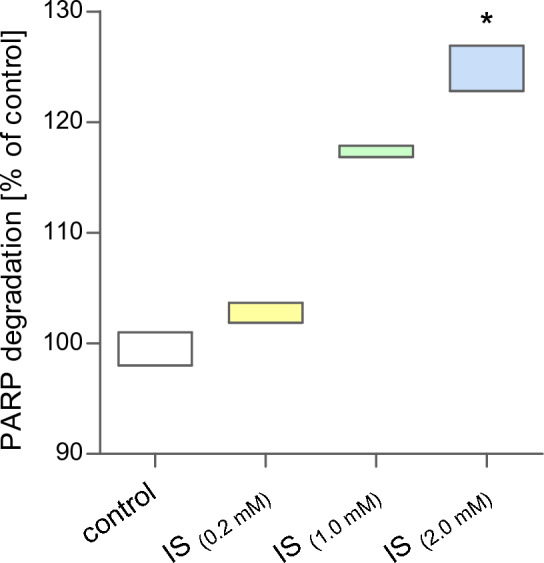
The percent of PARP degradation in mononuclear blood cells exposed to indoxyl sulfate. Each column represents cells from 2 different individuals (n = 2). Data were presented as a box of minimum and maximum values, a mean value of PARP degradation in control cells was arbitrarily taken as 100%, * *p* < 0.05—IS_(0.2 mM)_ versus control.

Poly(ADP-ribose) polymerase (PARP) enzymes are broadly involved in the cellular response to DNA damage. The high activity of PARP depletes NAD+ cell reserves and induces ATP depletion by inhibiting the oxidation of glucose, which can lead to cell death. PARP may be inactivated by cleavage by caspase 3. The analysis of PARP degradation by ELISA kit showed that IS could influence the cleavage process in MNCs. The effect of IS on PARP cleavage is shown in Fig. 5. After incubation of MNCs with IS, a directly proportional correlation was observed between the degree of PARP cleavage and the used concentration of the compound. In cells treated with IS at concentrations of 0.2 mM and 1.0 mM, no statistically significant changes in the level of PAPR degradation were observed. However, a statistically significant increase of PARP cleavage (approximately 25%) was observed in cells treated with 2.0 mM of IS.

**Figure 6:**
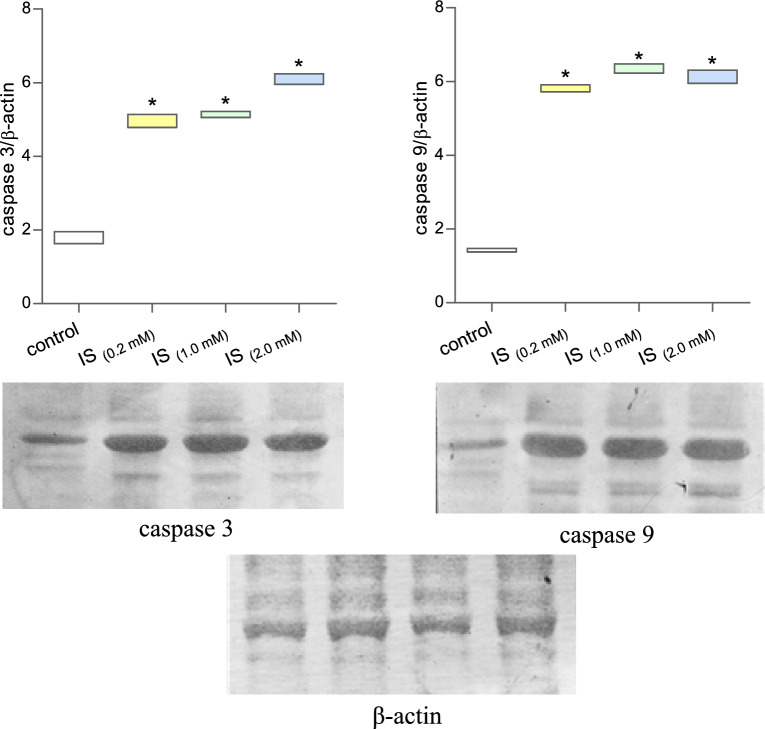
Expression of caspase 3 and 9 in mononuclear blood cells exposed to indoxyl sulfate. Each column represents cells from 3 different individuals (n = 3). Data were presented as a box of minimum and maximum value, *p < 0.05—IS_(0.2 mM)_, IS_(1.0 mM)_, and IS_(2.0 mM)_, versus control.

The induction of apoptosis in cells is related not only to the modification of the activity of various proteins but also to changes in their expression. The expression level of selected pro- and anti-apoptotic proteins in MNCs exposed to indoxyl sulfate was determined. The obtained results showed a significant increase in the expression of both caspase 3 and caspase 9 in cells treated with indoxyl sulfate (Fig. 6, Supplementary Information 1, 4, 5). It is worth noting that the expression of both of these proteins significantly increased right after exposing the cells to the lowest of investigated concentration of IS. The concentration of 0.2 mM of IS was determined as the average observed in patients with chronic kidney disease.

**Figure 7:**
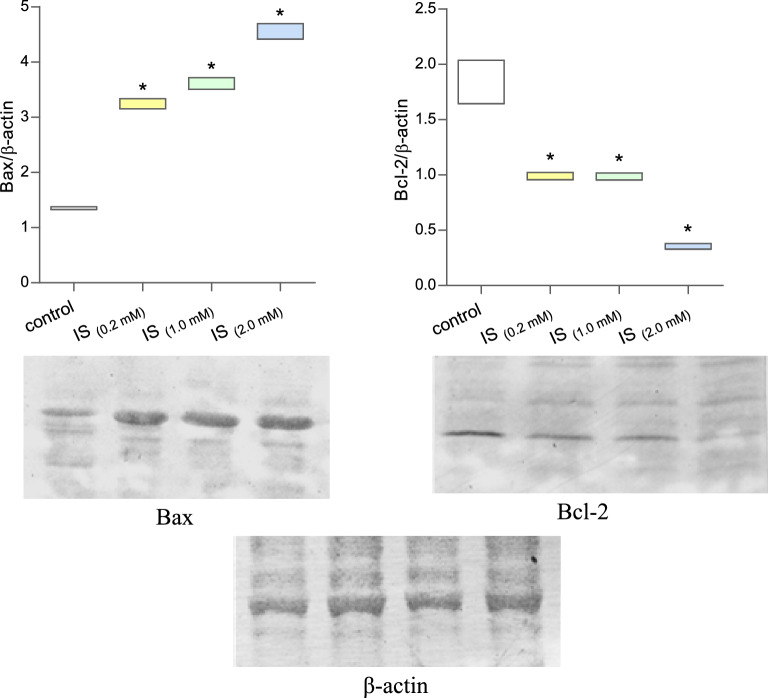
Expression of pro-apoptotic (Bax) and anti-apoptotic (Bcl-2) proteins in mononuclear blood cells exposed to indoxyl sulfate. Each column represents cells from 3 different individuals (n = 3). Data were presented as a box of minimum and maximum value, *p < 0.05—IS_(0.2 mM)_, IS_(1.0 mM)_, and IS_(2.0 mM)_, versus control.

The apoptosis process is associated with many proteins' activation and/or inhibition, such as the pro-apoptotic protein Bax and the anti-apoptotic protein Bcl-2. In the paper, the expression of these two proteins was analyzed using the Western Blot technique. A significant increase in Bax protein expression was observed in IS-treated MNCs (Fig. 7, Supplementary Information 1, 2, 3). Importantly, an approximately twofold increase in Bax protein expression was observed after incubating cells with IS at a 0.2 mM concentration—described as the average value in CKD patients. In the case of the Bcl-2 protein in MNCs incubated with IS, a significant decrease in expression was observed compared to the values for control cells (Fig. 7, Supplementary Information 1, 2, 3). Interestingly, IS at a concentration of 0.2 and 1.0 mM decreased Bcl-2 expression in MNCs by about half, and at a concentration of 2.0 mM about 4 times compared to the control.

## Discussion

Indoxyl sulfate is one of the uremic toxins which concentration in CKD patients increases over the average more than 80 times compared to healthy people. Regarding the levels seen in CKD patients, IS ranks third after guanidinosuccinic acid and methylguanidine^1,2^. It should therefore be assumed that this compound is of great importance in the proper functioning of the body.

The studies showed a significant decrease in the survival of MNCs treated with IS at a concentration of 2.0 mM. The cytotoxicity of IS measured using neutral red is in agreement with previously obtained IS cytotoxicity results XTT (tetrazolium salt reduction) assay^11^. Similar levels of indoxyl sulfate cytotoxicity were also observed against the kidney mesangial cell line (CRL-2573)^16^, murine C2C12 cell line^17^, primary human kidney PTEC and HK-2 cells, an immortalized human PTEC line^9,15^. However, other studies have shown that IS significantly reduces the survival of HepG2 (human liver carcinoma cells) and THLE-2 (immortalized human normal liver cells) at a concentration of 2.5 mM^13^.

The neutral red assay is a colorimetric assay measuring the uptake of the dye by functional lysosomes in living cells, whereas the MTT or XTT assay is mainly based on the enzymatic conversion of MTT/XTT in the mitochondria. The metabolic activity of cells in these areas may be different, and thus the obtained results of cytotoxicity measured by these two methods may be slightly different. The authors of comparative studies of different methods of cytotoxicity determination suggest the use of more than one method to determine cell viability in in vitro studies, to increase the reliability of the results obtained^18^. Indoxyl sulfate is a molecule that has a high affinity for proteins. In plasma, albumin is a protein that binds this compound very strongly. The tests on HK-2 cells showed that the albumin concentration in growing media did not affect IS-induced changes in metabolic activity^15^.

IS is believed to be one of the most biologically active uremic toxins directly involved in the pathogenesis and course of chronic kidney disease^19^. The biological activity of IS during chronic kidney disease is correlated with the severity of numerous pathological conditions, including the induction of oxidative stress, inflammation, and activation of signalling pathways related to NF-κB, p53, STAT3, TGF-β and Smad2/3^20–22^. The pro-oxidative properties of IS are related to the IS-dependent generation of reactive oxygen species H_2_O_2_, HO˙ and O_2_˙^−^, as well as depletion of the glutathione pool and redox homeostasis disturbances. Research shows that IS-dependent induction of oxidative stress often leads to cell death by apoptosis^9,13,23^. Recent studies by Ellis et al. indicate that IS plays a crucial role in the induction of programmed renal tubular cell death in ischemic or toxic kidney damage^15^. Similarly, in recent in vitro studies using the human hepatocyte model, Deng et al.^13^ emphasize the role of IS in inducing apoptosis of these cells. Uremic toxin generated oxidative stress and subsequent mitochondrial dysfunction, increasing Bax (BCL2 Associated X, Apoptosis Regulator)/Bcl-2 (B-cell leukemia/lymphoma 2 protein) levels and caspase-3 and -9 activity. It is worth emphasizing that IS-dependent apoptosis occurs primarily in the mitochondrial pathway.

In order to assess the pro-apoptotic properties of IS in MNCs, we analyzed the activities and expressions crucial for this process caspases and proteins from the Bcl-2 family: pro-apoptotic Bax and anti-apoptotic Bcl-2. Valuable results are provided by the analysis of the activity of cysteine proteases—caspase-2, -3, -6, -8 and -9 in MNCs treated with IS. Protein activity was assessed by spectrophotometric ELISA, and additionally, in the case of caspase-3 and -9, Immunoblotting expression analysis was performed with the simultaneous use of densitometric analysis. Our results are in line with previous studies where IS induced the activation of caspases involved in the mitochondrial apoptosis pathway^16,23^. Exposure of MNCs to IS led to a statistically significant increase in caspase-9 and -3, which are the initiator and executive caspases of the intrinsic PCD pathway, respectively. The increase in the activity of these caspases was confirmed by us both in ELISA and Western Blot tests, where an approximately two–threefold increase in their expression was observed compared to control cells (densitometric results were compared to the actin reference protein). Interestingly, in the case of caspase-2, the ELISA analysis showed a statistically significant increase in the protein already at the lowest IS concentration used (0.2 mM). The role of caspase-2 in the course of apoptosis has yet to be fully understood. It is known, however, that it is involved in cell death induced by, e.g., DNA damage and reactive oxygen species. Moreover, caspase-2 may be a molecular link between the mitochondrial and apoptosis receptor pathways by activating the pro-apoptotic protein BH3-only Bid from the Bcl-2 family^24^. On the other hand, as our studies indicate, IS did not cause statistically significant changes in the activity of caspase-6 and -8 in MNCs. The effector caspase-6 is primarily involved in neuronal cell death in neurodegenerative diseases^25^. To our knowledge, the available scientific literature does not indicate IS-dependent activation of the mentioned protease, especially in the pathogenesis of CKD. In turn, the lack of increase in the activity of caspase-8 in MCNs treated with IS confirms the hypothesis of apoptosis induction by this uremic toxin, primarily in the mitochondrial (internal) pathway, in which this protease is not directly involved.

Since apoptotic cell death induced by IS occurs mainly in the mitochondrial pathway, we analyzed the activity of two key representatives of the heterogeneous Bcl-2 protein family—the anti-apoptotic Bcl-2 peptide and the pro-apoptotic Bax, in addition to the caspases. Proteins belonging to the Bcl-2 family are essential regulators of the apoptosis process. For the biological fate of the cell, the ratio of gene expression levels of anti- and pro-apoptotic proteins is significant. Among others, Bcl-2 and Bax. Bcl-2 is the major member of the multidomain Bcl-2 subfamily. Its primary function is the inhibition of apoptosis in cells. This mechanism is accomplished by heterodimerization with pro-apoptotic members of the Bcl-2 family and disabling their apoptogenic properties. Bax belongs to the multidomain, pro-apoptotic subfamily of Bax proteins. The major pool of unactivated Bax polypeptide resides in the cytosol. Upon activation of Bax, dependent on the recognition of apoptotic signals and the participation of BH3-only polypeptides, it is translocated within the membranes of these organelles. Through its channel-forming activity in lipid bilayers, this polypeptide releases apoptogenic particles from mitochondria (including cytochrome c, Smac/DIABLO), disrupting the integrity of these organelles and eventual cell death^26,27^.

Immunoblotting analysis of Bax/Bcl-2 protein expression in MCNs treated with IS showed a clear and significant increase in the activity of the pro-apoptotic Bax peptide, with a simultaneous decrease in the anti-apoptotic partner Bcl-2. The increase in the Bax/Bcl-2 activity ratio was correlated with the IS concentration used. Given the results of IS-dependent apoptosis induction, it is reasonable to conclude that the increase in Bax, combined with reduced mitochondrial function, is the primary mechanism of programmed death in response to IS in human MCNs. Our findings of the pro-apoptotic properties of IS and the induction of programmed cell death, primarily in the mitochondrial (intrinsic) pathway, are consistent with previous studies. Notably, as outlined by Park et al.^9^ and Ellis et al.^15^, IS in human renal proximal tubular epithelial (HK-2) cells and human kidney proximal tubular epithelial cells (PTECs) induced apoptosis in the mitochondrial dish. IS treatment decreased Bcl-2 expression and increased the expression of apoptosis-related protein Bax. Moreover, as indicated by the studies by Kim et al.^23^, IS caused an increase in the activity of caspases 3/7 in the cultured osteoblast cell line MC3T3-E1 and ultimately led to their apoptosis. Pro-apoptotic mechanisms of IS activity in patients with CKD often play an important role in the pathogenesis of the disease. The latest reports of the team of Duangchan et al.^28^ emphasize the crucial role of the apoptogenic properties of IS in the development of CKD. As the authors indicate, IS-dependent apoptosis of erythrocytes and their senescence significantly impair the differentiation capacity of erythrocytes and impair erythropoiesis. This may be one of the leading causes of anaemia in patients with chronic kidney disease.

An inherent element accompanying apoptosis is disrupting the integrity and functionality of the mitochondria of cells affected by programmed death. An important factor affecting the metabolic pathways of cells is the proper functioning of mitochondria. Structural and functional disorders of the components of the mitochondrial membranes may contribute to changes in the potential of the mitochondrial membrane and, consequently, to the impairment of their function. In our work, we observed decreased mitochondrial membrane potential in MNCs treated with IS. It is worth emphasizing that indoxyl sulfate at the concentration already observed in CKD patients (0.2 mM) caused a significant decrease in the mitochondrial potential of cells by about 20% compared to the control values. The study of the MNCs of patients with end-stage kidney disease (ESKD) and healthy controls showed significant differences in mitochondrial profiles^29^. Significant changes in the membrane potential of mitochondria under the influence of IS in mouse C2C12 myoblast cells were also observed^14^. Moreover, in the same cells treated with IS, it has been shown that a decreased expression of muscular PCG-1α is a master regulator for mitochondrial biosynthesis. The authors of this paper suggest that reactive oxygen species formed as a result of IS activity may be responsible for mitochondrial dysfunction in cells. Previous works show an increased level of reactive oxygen species in cells exposed to IS^9,11,12^.

The next step in assessing the molecular mechanisms of IS biological activity in MNCs was the analysis of genotoxic properties, which are undoubtedly related to the apoptosis process. In addition to the undoubted role of NF-κB in shaping the genotoxicity of IS, the key to IS-dependent generation of DNA damage may be oxidative stress and ROS^30,31^. Oxidative stress is a common denominator in the pathogenesis of CKD^32,33^. Research shows that IS has shown pro-oxidant effects in various organs and cell types^13,34–37^. Emerging evidence from experimental studies reveals that IS contributes to the progression of CKD, possibly through increased oxidative stress and DNA damage^30,31^. Our results in the alkaline version of the comet test indicate the significant genotoxic potential of IS, which is a valuable scientific confirmation of previous results. It is worth noting that for the lowest concentration of IS (0.2 mM), no statistically significant DNA damage was observed in MCNs; however, already at the concentration of 1 and 2 mM, these changes were significant and reached approximately 10% and 20%, respectively, compared to control cells. Interestingly, Armand et al., in their latest work, indicate the lack of DNA damage generated by IS in the human colonic epithelial HT-29 and Caco-2 cell lines^38^. This may suggest the complexity of the molecular mechanisms of IS biological activity in cells of various origins.

A valuable supplement to the studies on the genotoxic properties of IS is the ELISA analysis of the degradation of the Poly(ADP-ribose) polymerase (PARP) protein. To our knowledge, our in vitro studies, for the first time, provide information on the effect of IS on said protein and its activity. Poly-ADP-ribose polymerases (PARPs) are a complex family of nuclear proteins with enzyme properties involved in primary cell life processes, such as maintaining genomic stability, repairing damaged DNA, and cell death. PARPs are activated by DNA strand breaks and play a key role, especially in the repair of single-strand breaks. Inhibition of PARP or their degradation contributes to limiting the effective repair of DNA, intensifying mutations and genetic instability, and thus, in consequence, cell death. Moreover, the activation of caspase-3, followed by PARP cleavage, is a key event in the process of apoptosis^39,40^. Statistically significant IS-dependent degradation of PARP in MCNs was observed at the highest concentration of 2 mM. An approximately 20% increase in PAPR degradation may indicate inhibition of DNA damage repair and activation of apoptosis in MNCs exposed to IS. Notably, these results align with our analysis of IS-dependent induction of DNA damage (comet assay) and activation of caspases (including caspase-3 and -9) in MNCs.

## Conclusion

The progression of chronic kidney disease is related mainly to the accumulation of uremic toxins in the body. Indoxyl sulfate is a toxin that strongly interacts with proteins, which in turn can lead to changes in their structure and function. The conducted studies showed that this compound can induce the programmed death of MNCs through the internal mitochondrial apoptosis pathway. The observed cellular changes are mostly dose-dependent. The search for effective IS absorbents could slow the progression of CKD and improve patients' quality of life.

## Material and methods

### Chemicals

Indoxyl sulfate potassium salt (IS), RIPA buffer, secondary antibodies conjugated with alkaline phosphatase, 5,5′,6,6′-Tetrachloro-1,1′,3,3′tetraethylbenzimidazolocarbocyanine iodide (JC-1), 4′,6-Diamidino-2-phenylindole dihydrochloride (DAPI), 3-Amino-7-dimethylamino-2-methylphenazine hydrochloride (Neutral red), LMP agarose type XI, NMP agarose type I were purchased from Sigma-Aldrich (Sigma-Aldrich Sigma, St. Louis, MO, USA). Mouse polyclonal antibodies for Bax, Bcl-2, caspase-3, caspase-9 and β-actin were obtained from Santa Cruz Biotechnology (USA). PARP Degradation ELISA—PARP (Cleaved) [214/215] Human ELISA Kit was purchased from Thermo Fisher Scientific (MA USA). Unless otherwise indicated, all other chemicals were purchased from POCH S.A. (Gliwice, Poland). All dishes necessary for cell culture were obtained from NUNC.

### Isolation of mononuclear blood cells (MNCs)

The research was conducted on mononuclear blood cells (MNCs) isolated from the human blood buffy coat obtained from the Regional Center for Blood Donation and Haemotherapy (RCKiK) in Lodz, Poland. Every single experiment was performed at least in duplicate (3–4 times) on cells from one donor, and n-numbers of presented results represent cells from different individuals.

Cell isolation was performed by density gradient centrifugation of blood buffy coat with Histopaque®-1077 (Sigma-Aldrich Sigma, St. Louis, MO, USA). The isolated MNCs were washed two times with phosphate-buffered saline pH 7.4 (PBS).

### Cells treatment conditions

Freshly isolated mononuclear blood cells (MNCs) were placed in RPMI medium (Corning, Mediatech, Inc, Manassas, VA, USA) supplemented with 10% heat-inactivated foetal bovine serum (Capricorn Scientific, GmbH) and antibiotics 10 U/mL penicillin and 50 μg/mL streptomycin (Corning, Mediatech, Inc, Manassas, VA, USA).

Cells were seeded at suitable density for the performed assay into 96-well plates (5 × 10^4^) or Petri dishes (4 × 10^6^) and incubated for 24 h with indoxyl sulfate (IS) at a final concentration of 0.2 mM, 1 mM 2 mM in standard culture conditions (37 °C, 100% relative humidity, 5% CO_2_ incubator). Indoxyl sulfate was dissolved in PBS. Control cells were treated with a corresponding volume of PBS. After incubation, cells were washed with PBS and used for the following measurements.

### The cytotoxicity test

The Neutral Red Uptake (NRU) method is a cytotoxicity test based on the uptake and accumulation of neutral red inside organelles with an acidic pH. The transport of the dye takes place in a passive way. Neutral red penetrates a living cell with an intact membrane cell and accumulates inside lysosomes and endosomes. The amount of neutral red accumulated in the organelles is proportional to the number of living cells^41^.

After incubation, the cells were placed for 2 h in a medium containing neutral red. Subsequently, the cells were washed, the dye was extracted in each well, and the absorbance was measured at a wavelength of λ = 540 nm on a microplate reader (BioTek). The obtained absorbance values of the reaction product were presented as a percentage, taking the absorbance value of the control as 100%.

### Caspase activity test

The activities of caspases were determined using Life Technologies™ ApoTarge™ caspase colourimetric protease assay sampler kit (caspases-2, -3, -6, -8, and -9). According to the information provided by the manufacturer, the assay contains the substrates VDVAD (for caspase-2), DEVD (for caspase-3), VEID (for caspase-6), IETD (for caspase-8) and LEHD (for caspase-9. The substrates for caspase activity measurement are labelled at their C-termini with para-nitroaniline (pNA). The light absorption by free pNA can be measured at 400 or 405 nm. Comparing the absorbance of pNA from samples allows for determining changes in caspase activity. The experiment was performed according to the manufacturer's protocol and as described before^42^. The results are presented as a percentage of change in absorbance at 405 nm relative to control calculated as 100%.

### Measurement of mitochondrial membrane potential

The mitochondrial potential in MNCs was measured using a JC-1 fluorescent probe^43^. This fluorescent carbocyanine dye accumulates in the mitochondrial membrane in two forms (monomers and dimers). The negative potential of the inner mitochondrial membrane facilitates the formation of dye aggregates. Measuring the ratio of JC-1 dimer to monomer fluorescence is a suitable method for estimating changes in mitochondrial membrane potential.

After the treatment, the cells were placed in Hank’s buffered salt solution (HBSS) containing 5 µmol/L JC-1 fluorescent probe and incubated in total darkness for 30 min at 37 °C. Before fluorescence measurements, cells were washed to remove the dye and suspended in fresh HBSS solution. The fluorescence intensity of the monomer and dimer was measured on a spectrofluorometer Cary Eclipse (Varian, Inc.) using filter pairs of 535 nm/590 nm (dimers) and 475 nm/530 nm (monomers). Subsequently, the ratio of the fluorescence intensities at 530 nm/590 nm was calculated^42^. The mitochondrial potential in MNCs was measured immediately after incubation with indoxyl sulfate. Results are expressed as a percentage of the control, which was taken as 100%.

### Measurement of DNA fragmentation

DNA damage was determined by comet assay (single-cell electrophoresis), a rapid and sensitive method for detecting single-stranded and double-stranded DNA breaks.

After 24 h of treatment with indoxyl sulfate, the cells were suspended in PBS 2.5 × 10^5^ cells/ml. Low melting point agarose (LMP type XI) was added to the cell suspension, and the mixture was applied to a glass slide previously coated with standard melting point agarose (NMP type I). After solidification of the agarose, glass slides were incubated in lysis buffer containing (2,5 mol/L NaCl, 100 mmol/L Na_2_-EDTA, 10 mmol/L TRIS, 1% Triton X-100) for 2 h. At the end of lysis, the electrophoresis was conducted in a buffer containing (1 mmol/L Na_2_-EDTA, 300 mmol/L NaOH) and (29 V, 30 mA). Before analysing, the slides were stained with DAPI solution (2 ng/ml)^42^. The analysis was performed under alkaline conditions according to the procedure of Singh et al.^44^ with slight modifications. The slides were analysed using a fluorescence microscope Zeiss Axio Scope A1 (Carl Zeiss, Germany) with a video camera connected to the image-analysis system Lucia-Comet v. 7.60 (Laboratory Imaging, Praha, Czech Republic). The values are the mean ± SD of 50 cells from glass slide in 6 independent experiments.

### Cell lysate and immunoblotting

In order to assess the expression of proteins (caspase-3, caspase-9, Bax, Bcl-2 and β-actin), MNCs after incubation cells were lysed (4 °C, 20 min) in a RIPA buffer containing 150 mM NaCl, 1.0% IGEPAL® CA-630, 0.5% sodium deoxycholate, 0.1% SDS, 50 mM Tris, pH 8.0 with protease inhibitors/EDTA (final concentration 10 µM). After centrifugation, the supernatants were collected^11^. About 30 µg of proteins were loaded into each lane. The probes were electrophoretically separated by 12.5% sodium dodecyl sulfate–polyacrylamide gel electrophoresis (SDS-PAGE) and transferred to Immobilon P as described by Towbin et al.^45^. Protein concentration in MNCs lysates was determined using the method of Lowry et al.^46^. Subsequently, the membranes were blocked in 5% nonfat dry milk in TBST buffer (10 mM Tris–HCl, pH 7.5, 150 mM NaCl, and 0.05% Tween 20) for 1 h at room temperature. After blocking, the membranes were incubated overnight with antibodies specific to Bax, Bcl-2, caspase-3, caspase-9 and β-actin (1:500 dilution) in a TBST buffer in a cold room. After 24 h incubation with the primary antibody, the membranes were washed thrice with TBST and incubated with appropriate secondary anti-mouse antibodies conjugated with alkaline phosphatase in TBST for 2 h at room temperature. The membranes were then washed several times with TBST. The proteins were visualized by incubation with the substrate solution (0.33 mg/mL of nitro blue tetrazolium, 0.17 mg/mL of 5-bromo-4-chloro-3-indolyl phosphate in 100 mM Tris–HCl, pH 9.5, 100 mM NaCl and 5 mM MgCl_2_), prepared according to Leary et al.^11,47^. Analysis of the relative change in the expression of the tested proteins was performed on the basis of signal intensity and densitometric analysis in ImageJ 1.48v software (National Institutes of Health, Bethesda, MD). The results are presented as the ratio of the densitometric values of the band intensity measurements for an investigated protein to the actin reference protein for each well.

### Measurement of cleaved PARP levels

After incubation, the cells were centrifuged (1500 rpm; 3 min; 4 °C) and the supernatant was removed. The collected cell pellet was lysed in RIPA buffer (50 mM Tris–HCL, pH 8.0, with 150 mM sodium chloride, 1.0% IGEPAL® CA-630 (NP-40), 0.5% sodium deoxycholate and 0.1% sodium dodecyl sulfate) with protease inhibitor (phenylmethylsulfonyl fluoride) (Sigma-Aldrich Sigma, St. Louis, MO, USA). Cleaved Poly (ADP-ribose) polymerase (PARP) levels were assessed using the PARP Cleaved [214/215] ELISA kit (Thermo Fisher Scientific, MA USA) according to the protocol described in the manufacturer's instructions and, as described before^48^.

### Statistical analysis

The normality of data was tested using the Shapiro–Wilk test, and variance homogeneity was verified with Levene’s test. The significance of the differences between the groups was estimated by a one-way ANOVA using Tukey’s post hoc multiple comparisons test for the data with normal distribution. For the data with a departure from normality, the non-parametric Kruskal–Wallis test was used. Statistical significance was accepted at *p* < 0.05. The sample size was estimated for type I and type II statistical errors of 0.05 and 0.8, respectively. Accurate conclusions were not formulated for data with statistical power below 80%. The statistical analysis was performed using Statistica v. 13.3 (StatSoft Polska, Krakow, Poland).

### Supplementary Information

Supplementary Information 1.

Supplementary Information 2.

Supplementary Information 3.

Supplementary Information 4.

Supplementary Information 5.

## Data Availability

The data presented in this study are available on request from the corresponding author.
