# Supplementary figures and images for: Indoxyl sulfate induces apoptosis in mononuclear blood cells via mitochondrial pathway
Source: Sci Rep. 2023 Aug 28;13:14044. doi: 10.1038/s41598-023-40824-z (PMC10462746; doi:10.1038/s41598-023-40824-z)

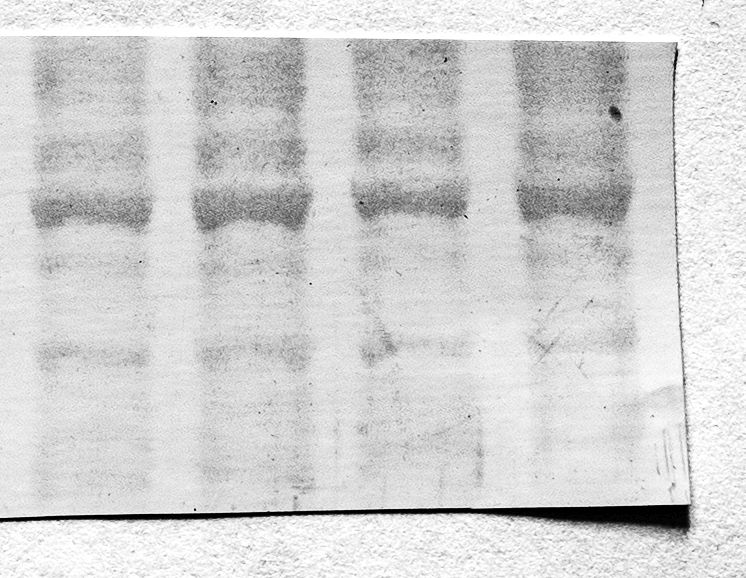

Supplement: Supplementary file 1 — Supplementary Information 1. [file 41598_2023_40824_MOESM1_ESM.jpg]

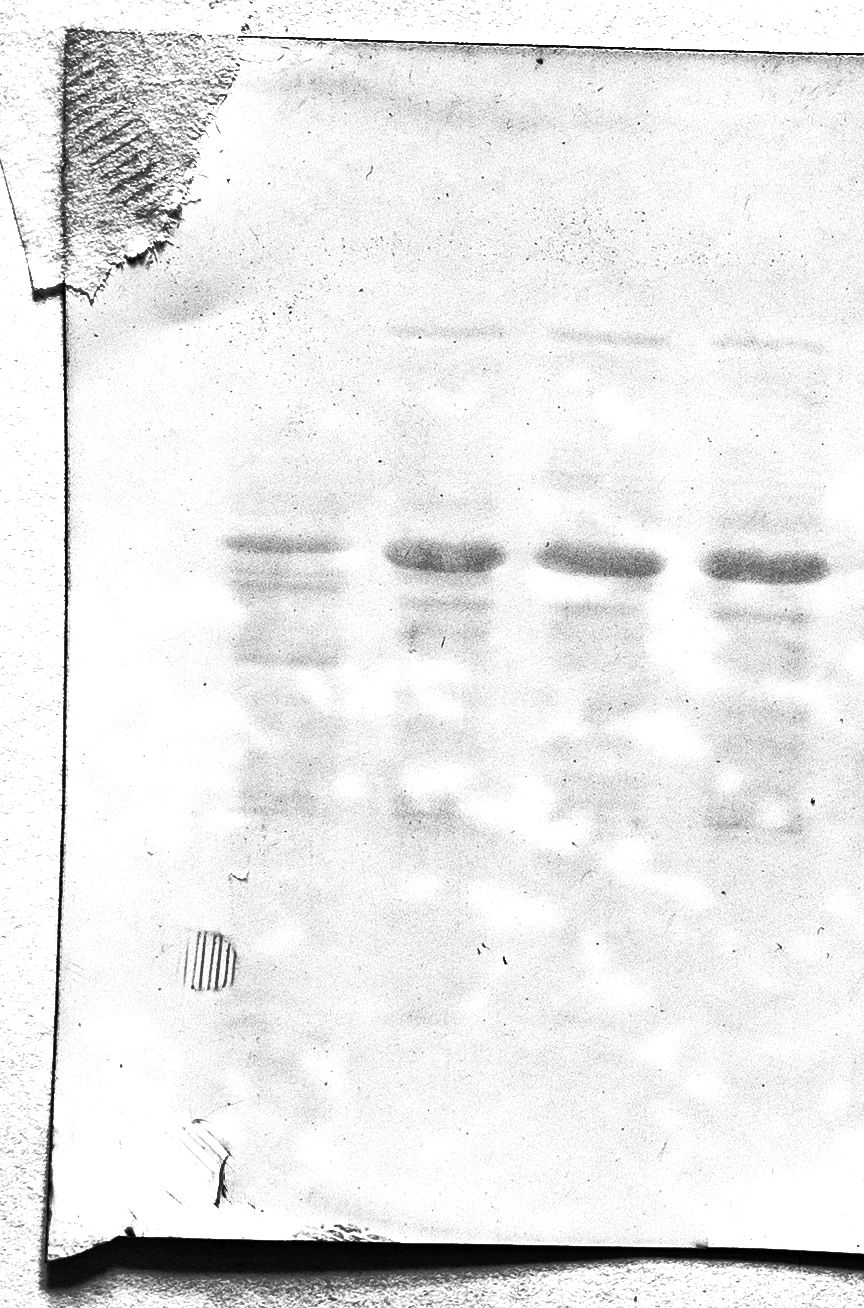

Supplement: Supplementary file 2 — Supplementary Information 2. [file 41598_2023_40824_MOESM2_ESM.jpg]

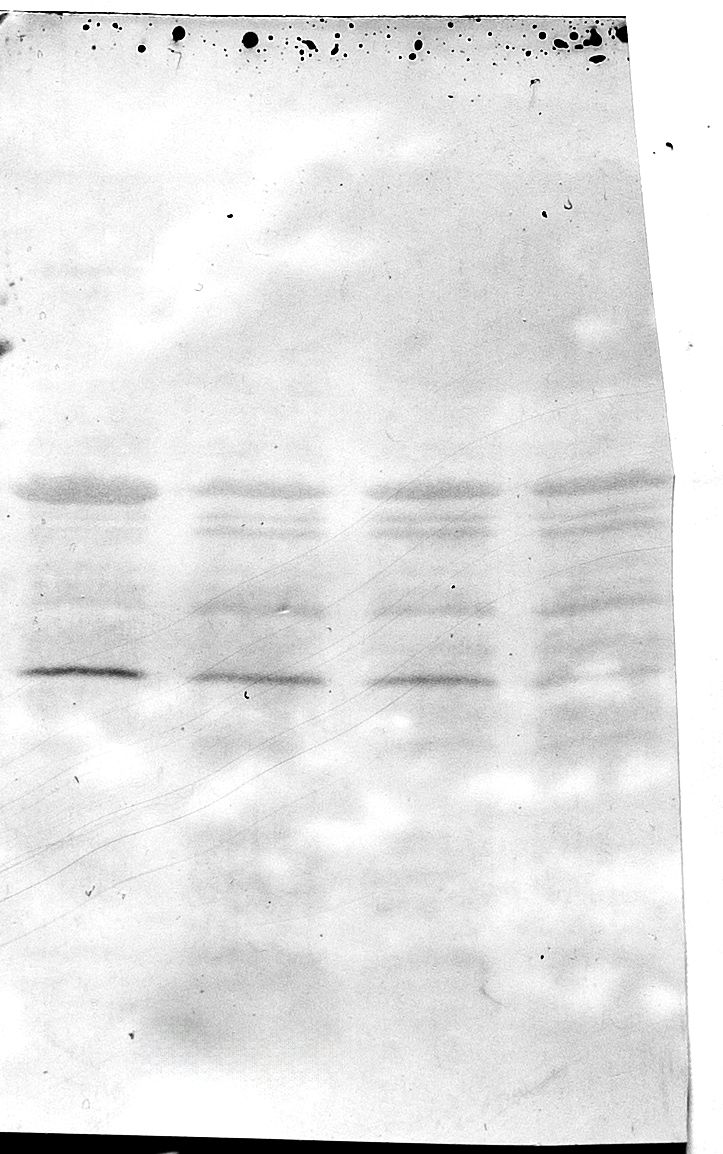

Supplement: Supplementary file 3 — Supplementary Information 3. [file 41598_2023_40824_MOESM3_ESM.jpg]

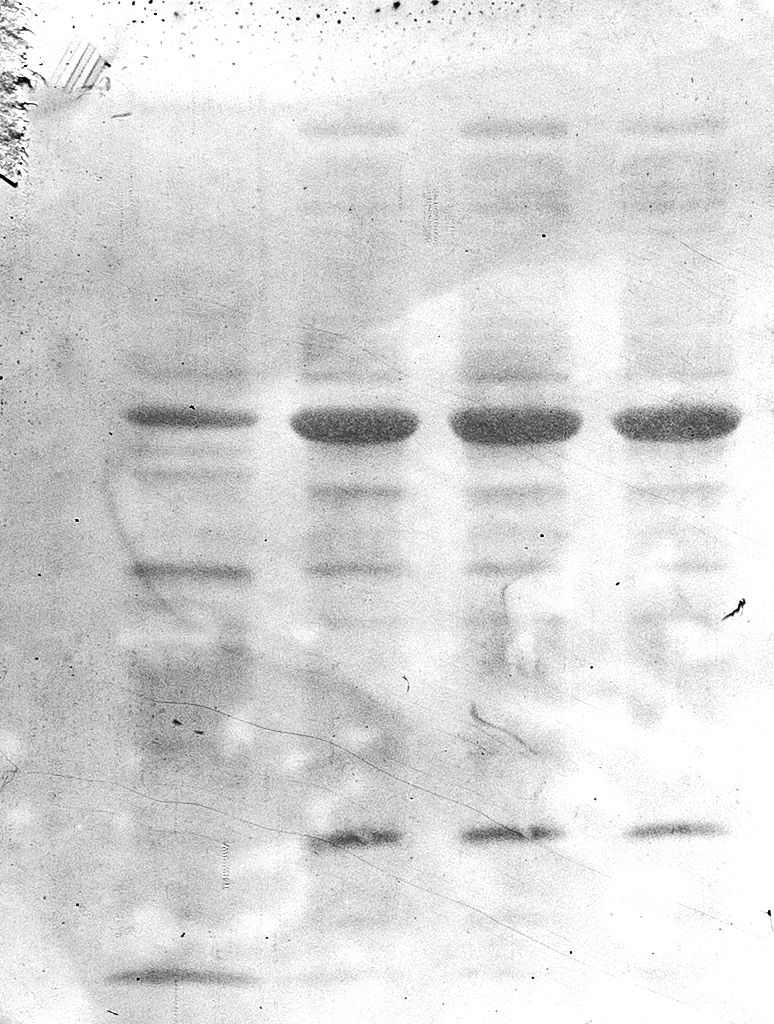

Supplement: Supplementary file 4 — Supplementary Information 4. [file 41598_2023_40824_MOESM4_ESM.jpg]

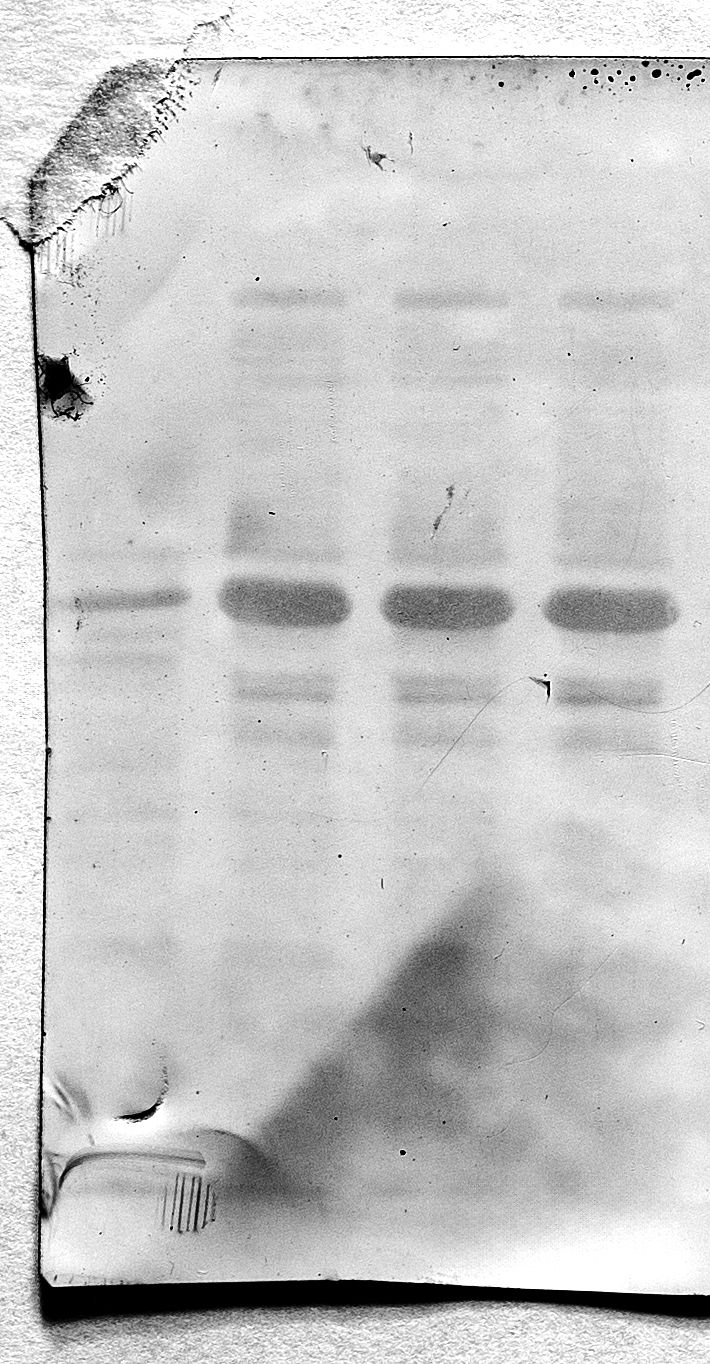

Supplement: Supplementary file 5 — Supplementary Information 5. [file 41598_2023_40824_MOESM5_ESM.jpg]
